# Human *Erysipelothrix rhusiopathiae* infection *via* bath water – case report and genome announcement

**DOI:** 10.3389/fcimb.2022.981477

**Published:** 2022-10-24

**Authors:** Andreas E. Zautner, Aljoscha Tersteegen, Conrad-Jakob Schiffner, Milica Ðilas, Pauline Marquardt, Matthias Riediger, Anna Maria Delker, Dietrich Mäde, Achim J. Kaasch

**Affiliations:** ^1^ Institut für Medizinische Mikrobiologie und Krankenhaushygiene, Medizinische Fakultät der Otto-von-Guericke Universität Magdeburg, Magdeburg, Germany; ^2^ Universitätsklinik für Plastische, Ästhetische und Handchirurgie Medizinische Fakultät der Otto-von-Guericke Universität Magdeburg, Magdeburg, Germany; ^3^ Landesamt für Verbraucherschutz Sachsen-Anhalt, Halle (Saale), Germany

**Keywords:** *Erysipelothrix rhusiopathiae*, case report, genome, Vancomycin Resistance, erysipeloid, swine erysipelas, MSMR, vex23-vncRS

## Abstract

*Erysipelothrix rhusiopathiae* is a facultative anaerobic, environmentally stable, Gram-positive rod that causes swine and avian erysipelas as a zoonotic pathogen. In humans, the main manifestations described are circumscribed erysipeloid, generalized erysipeloid, and endocarditis. Here, we report a 46-year-old female patient who presented to the physician because of redness and marked *functio laesa* of the hand, in terms of a pain-related restricted range of motion, and was treated surgically. *E. rhusopathiae* was detected in tissue biopsy. The source of infection was considered to be a pond in which both swine and, later, her dog bathed. The genome of the isolate was completely sequenced and especially the presumptive virulence associated factors as well as the presumptive antimicrobial resistance genes, in particular a predicted homologue to the multiple sugar metabolism regulator (MsmR), several predicted two-component signal transduction systems, three predicted hemolysins, two predicted neuraminidases, three predicted hyaluronate lyases, the surface protective antigen SpaA, a subset of predicted enzymes that potentially confer resistance to reactive oxygen species (ROS), several predicted phospholipases that could play a role in the escape from phagolysosomes into host cell cytoplasm as well as a predicted vancomycin resistance locus (*vex23-vncRS*) and three predicted MATE efflux transporters were investigated in more detail.

## Introduction

A 46-year-old female patient presented to our clinic with swelling, redness, and pain on the right thumb that had been progressive for two days. The patient reported that she suffered a minor lesion of the thumb pad from cracking walnuts. On inspection, a blister approximately 1 cm in diameter was found on the palmar end phalanx of the right thumb, still covered by intact skin. A marked swelling with semicircular redness extended down to the proximal phalanx. On the extensor surface, a well-demarcated striated lymphangitis reached across the wrist down to the distal forearm. Clinical inspection did not allow a clear distinction between erysipelas and cellulitis. To explore, whether a foreign body may have remained from walnut cracking, an incision was made under local anesthesia, which revealed no pus, no indurated tissue, and no foreign body.

## Background

### The genus *Erysipelothrix*

*E. rhusiopathiae* belongs to the Erysipelotrichaceae family and is the only human pathogenic microbial species of the genus *Erysipelothrix*. Further microbial species of the genus have been described more recently and include *Erysipelothrix tonsillarum* (Takahashi et al., 1987), *Erysipelothrix inopinata* (Verbarg et al., 2004), *Erysipelothrix muris* (Chen et al., 2006), *Erysipelothrix larvae* (Bang et al., 2015; Bang et al., 2016), *Erysipelothrix piscisicarius* (Pomaranski et al., 2020), *Erysipelothrix anatis* sp. nov., *Erysipelothrix aquatica* sp. nov., *Erysipelothrix urinaevulpis* (Eisenberg et al., 2022) and the as yet undesignated *Erysipelothrix* species 1, *Erysipelothrix* species 2, and *Erysipelothrix* species 3 (Takahashi et al., 2008).

### Historical classification of the species *Erysipelothrix rhusiopathiae*

The species designation changed a number of times. Robert Koch first isolated a bacterium of the genus *Erysipelothrix* in 1876 from a mouse that he had previously inoculated with putrid blood. He designated this pathogen as the bacterium of mouse septicemia, *E. mursiseptica* (Wang et al., 2010). Friedrich Löffler isolated a similar organism, under the name *Bacillus* of swine erysipelas (latinized by Kitt in 1893 as *Bacillus rhusiopathiae suis*), from the skin blood vessels of a pig that had died of swine erysipelas in 1886 and he was the first to describe the pathogen and the disease caused by it in swine (Wang et al., 2010). Friedrich Julius Rosenbach isolated a bacterium similar to Robert Koch’s bacterium from a patient with localized skin lesions in 1909, so that *Erysipelothrix* was now established as a human pathogen after initial case reports since 1870. Rosenbach coined the term “erysipeloid” to distinguish between the streptococcal infection “erysipelas” and the efflorescence he had observed. Rosenbach still distinguished three different microbial species: *E. muriseptica*, *E. porci*, and *E. erysiploides*, depending on their origin of isolation from mice, swine, or humans (Wang et al., 2010). Later it was discovered that they were three nearly identical isolates of the same microbial species, which was named *E. insidiosa*, as originally proposed by Trevisan in 1885. This name, as well as all other 36 names circulating for this bacterium at the time, were discarded in 1966 in favor of *E. rhusiopathiae*, a combination of genus and species that had been coined as early as 1918 (Wang et al., 2010). According to comparative genomic analyses, the species *E. rhusiopathiae* can be further subdivided into three clades, as well as a fourth clade phylogenetically intermediate between clades 2 and 3 (Forde et al., 2020). In addition, *Erysipelothrix* spp. can be divided into at least 28 known serovars (Opriessnig et al., 2020). Serovar 1 is considered to be the most virulent and therefore of greatest veterinary importance (Selbitz et al., 2011).

### Culture and morphology

**Figure 1:**
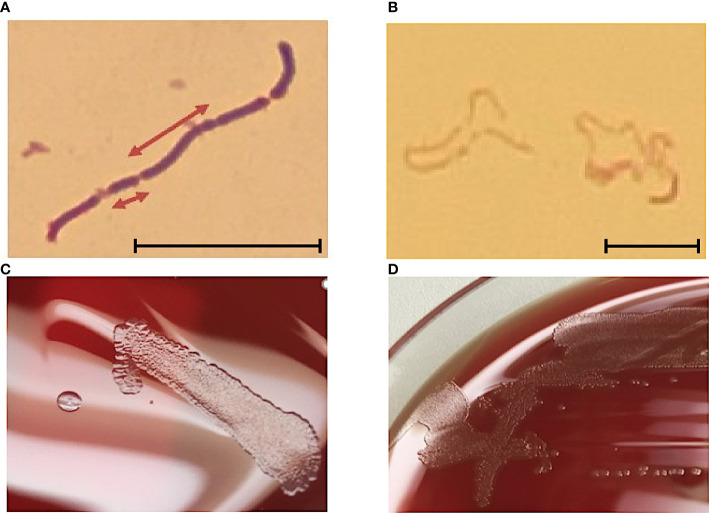
**(A)** *E. rhusopathiae* Gram stain (magnification 1:1000, scale bar = 5 µm). The arrows indicate two Gram-positive rods with distinctly different lengths. **(B)** Gram stained slide with *E. rhusopathiae* (magnification 1:1000, scale bar = 5 µm). A decolorized (pseudo-) Gram-negative sample is shown. This phenomenon occurs regularly when preparing the microscopic specimen with colonies appearing **“**rough**”** on agar plates. **(C)** Growth of *E. rhusopathiae* on Schaedler KV agar. On the left, a smooth clearly circumscribed pinpoint colony can be seen, next to a bed of confluent colonies (right). **(D)** smooth and rough colonies on Columbia sheep blood agar.

The cell wall of *E. rhusiopathiae* consists among others of lysine and glycine, which distinguishes it from *Listeria* and *Corynebacteria*. The negative catalase test differentiates *Erysipelothrix* spp. from *Kurthia* spp. In the Gram stain, Gram-positive rods are recognizable in chain formation, but with a highly variable single cell length, from 0.8 µm to 2.5 µm (**Figure 1A**). Occasionally, even filaments of up to 60 µm in length can be seen. *E. rhusiopathiae* is readily decolorized during Gram staining, and Gram-labile or even Gram-negative staining behavior may be apparent (**Figure 1B**) (Carroll et al., 2019).

Regarding culture conditions, *E. rhusiopathiae* is relatively undemanding and can be grown on aerobically incubated sheep blood or chocolate blood agar. Nevertheless, a capnophilic atmosphere favors bacterial growth. When grown on Schaedler KV agar, chocolate agar, or Columbia sheep blood agar, *E. rhusiopathiae* exhibits two colony morphologies. On the one hand, it appears as a smooth (S) colony in the form of clearly delimited pin-points, on the other hand, as a rough (R) colony with irregular boundaries (**Figures 1C, D**) (Carroll et al., 2019). The S-form is particularly observable in initial culture from acute disease cases, but the R-form is particularly observable in culture from chronic disease cases and after more frequent passaging *in vitro* (Selbitz et al., 2011). Moreover, the facultative anaerobic bacterium can be cultivated in trypticase soy or Schaedler broth. To suppress possible accompanying flora, especially when isolating *E. rhusiopathiae* from environmental and marine locations, or from animal waste and products, numerous selective media were developed (Brooke and Riley, 1999; Fidalgo et al., 2000; Wang et al., 2010).

### Identification

Recently, species identification of *E. rhusiopathiae* has become a minor issue with availability of MALDI-TOF mass spectrometry and modern biochemical methods such as VITEK^®^ 2 GP ID card (Biomérieux, Nürtingen, Germany), API^®^ Coryne (Biomérieux, Nürtingen, Germany), Rapid ID 32 STREP (Biomérieux, Nürtingen, Germany), or BD Phoenix™ PID Panel (BD Biosciences, Sparks, MD, USA). More reliable identification may have contributed to an observed increase of *E. rhusiopathiae* detection in human specimen since about 2008 (Farfour et al., 2012; Principe et al., 2016). Additionally, several PCR-based assays have been developed to detect *E. rhusopathiae*; to differentiate it from other *Erysipelothrix* species; and to subdifferentiate (serotype) within the microbial species (Fidalgo and Riley, 2004; Yamazaki, 2006; Pal et al., 2010; Shiraiwa et al., 2017; Shimoji et al., 2020). For epidemiological purposes, different subtyping methods have been developed based on multilocus sequence typing (MLST), pulsed-field gel electrophoresis (PFGE) (Janßen et al., 2015) and sequencing of the hypervariable region of the *spaA* gene (Nagai et al., 2008).

### Epidemiology

*E. rhusiopathiae* is widespread among mammals, birds, and fish, but most commonly found in swine and their feces (Funke, 2009). In approximately 50% of healthy swine, *E. rhusiopathiae* can be detected in the tonsils or lymphoid tissue (Spiteri and Taylor-Robinson, 2018). Due to its high tenacity, the pathogen persists for several months in soils and water bodies as well as in decaying animals and fish (Selbitz et al., 2011). Human infection usually occurs in occupational groups exposed to appropriate animal products or excreta, such as farmers, veterinarians, furriers, butchers, fishermen, fishmongers, homemakers, cooks, and grocers.

### Disease in swine and other animals

Susceptible to infection with *E. rhusiopathiae* are primarily swine between 3 and 12 months of age. After oral, conjunctival, or percutaneous infection, bacteremia occurs after three to five days of incubation, later resulting in the characteristic manifestations of the skin (Selbitz et al., 2011). Acute swine erysipelas, most commonly associated with serovar 1 - subtype 1a, manifests with high fever up to 42°C and the typical landmark redness of the skin. In the peracute form of the disease, the animals can also die before the pathognomonic symptoms have developed. This is referred to as “white erysipelas” (Selbitz et al., 2011). The subacute but also the acute course is characterized by pathognomonic raised rhomboid skin lesions of several centimeters in diameter that are called “diamond skin”. Chronicity of the symptoms of erysipelas, that this means a disease duration of more than four weeks, is possible. Chronic erysipelas may occur as a result of acute disease but may also be a direct consequence of persistent infection with low virulent strains of *E. rhusiopathiae*. Typical manifestations are skin necrosis, polyarthritides and/or *endocarditis valvularis*, which may be manifested by cauliflower-like thrombotic-ulcerative deposits on the atrioventricular valves (Selbitz et al., 2011). *E. rhusiopathiae* infections are not restricted to swine. It also occurs in sheep, in which the disease frequently manifests as chronic polyarthritis and rarely as septicemia, as well as in turkeys, ducks, chickens, mice, rats, beavers, cattle, rabbits, horses, minks, foxes, and dolphins (Selbitz et al., 2011). For prophylaxis, 17 different commercial vaccines are currently available in veterinary medicine (Opriessnig et al., 2020).

### Clinical manifestations in humans

In contrast to swine, serotypes 2, 7, and 16 are the most commonly involved in the pathogenesis of human erysipeloid (Veraldi et al., 2009). If the pathogen enters the skin *via* a wound, there are basically three clinical manifestations: a localized skin lesion (so-called erysipeloid), a generalized skin lesion, and bloodstream infection. Local erysipeloid is characterized by sharply circumscribed, painful, reddish, non-repressible edema often accompanied by vesiculation and erosive lesions without pus formation (Wang et al., 2010). In addition, arthralgia, myalgia, lymphadenitis and mild fever may occur (Rostamian et al., 2022). Frequently, the back of the hand (as in our patient) or the extensor side of the fingers are affected, because the tendons form a row being wrapped around very tight (Veraldi et al., 2009). Usually, the spread of the pathogen is limited to a roundish area at the point of entry. Rarely, the so-called multiple or systemic erysipeloid is reported. This results in the radial spread of multiple erysipeloids from the original focus (Wang et al., 2010) particularly seen in immunocompromised individuals (Veraldi et al., 2009). Bloodstream infection is rare but associated with severe disease, e.g. endocarditis (Drekonja, 2013; Hofseth et al., 2017; Wang et al., 2020). Other complications include meningitis, osteomyelitis, or septic arthritis (Wang et al., 2010; Groeschel et al., 2019). Immunosuppression represents an important causal factor for systemic infection.

## Methods

### Culture, species identification and susceptibility testing

According to the diagnostic routine at the Department of Medical Microbiology and Hospital Hygiene of the Medical Faculty of Otto-von-Guericke University Magdeburg, Columbia CNA agar with 5% sheep blood (aerobic), Schaedler/Schaedler KV agar (anaerobic) and Schaedler broth (media obtained from Fisher Scientific GmbH, Schwerte, Gemany) were inoculated with the tissue biopsy taken.

Species identification was performed both biochemically, using a VITEK^®^ 2 GP ID card and a VITEK^®^ 2 XL device (Biomérieux, Nürtingen, Germany) as well as by MALDI-TOF MS (Vitek-MS, Biomérieux, Nürtingen, Germany).

Susceptibility testing was carried out using MIC test strips (Liofilchem S.r.l., Roseto degli Abruzzi (Teramo), Italy).

### Whole genome sequencing

Prior to DNA isolation the *E. rhusiopathiae* isolate was cultivated on Columbia agar supplemented with 5% sheep blood (Becton Dickinson, Beckton-Dickinson, New Jersey, USA) and incubated at 37°C for 16h. DNA was isolated from agar colony material (ca. 5 C.F.U.) using the CTAB-lysozyme protocol by Larsen and coworkers (Larsen et al., 2007). Cells were harvested (10 min., 3,000 x g), resuspended in 450 µl GTE solution (10 mM EDTA and 50 mM Glucose in 25 mM Tris-CL) and digested for one hour at 37°C with lysozyme (adding 50 µl of 10 mg/ml lysozyme to a final concentration of 1 mg/ml). Then, the cell suspension was incubated after adding 150 µl proteinase K (10 mg/ml) in 10% SDS (30 min, 55°C). Treatment with 4 µl RNase A (Qiagen, Hilden Germany; 700 U/ml, 2 min, room temperature) was followed by addition of 200 µl NaCl (5 M). CTAB (4.1 g NaCl in 90 ml water + 10 g cetrimide, Sigma cat. No. H5882, in NaCl) was preheated to 65°C, 160 µl were added and the solution was incubated (10 min, 65°C). This is followed by 2 extraction steps of a chloroform:isoamyl alcohol extraction. After adding chloroform/isoamylalkohol (24:1, ca. 1 ml) the solution was centrifuged (10,000 x g, 5 min). Then the upper aqueous phase was transferred to a fresh tube and again chloroform/isoamylalkohol (24:1, ca. 0,9 ml) was added. The solution was centrifuged once again (10,000 x g, 5 min). The aqueous layer (800 µl) was transferred to a fresh tube, 560 µl isopropanol were added, and the tube was inverted until DNA precipitates. The suspension was incubated for 5 min at room temperature and subsequently centrifuged (10,000 x g, 10 min). Afterwards, the pellet was washed twice with ethanol (70%, 10,000 x g, 10 min). 50 µl TE-buffer were added after 15 min of air-drying. Library preparation was performed using the TruePrep DNA Library Prep Kit V2 for Illumina (1 ng) (Vazyme Biotech Co. Ltd., Nanjing, China) and samples were barcoded with the Nextera XT Index Kit (24 indexes, 96 samples, Illumina, San Diego, USA).

### Bioinformatics

Data were analyzed with Ridom SeqSphere+ (Ridom™, Münster, Germany) using a custom made core genome with the Fujisawa strain (NC_015601.1) as seed genome. Nine different *E. rhusiopathiae* genomes were used as query genomes (SY1027, NC_021354.1; GXBY-1, NZ_CP014861.1; WH13013, NZ_CP017116.1; ML101, NZ_CP029804.1; KC-Sb-R1; NZ_CP033601.1; NCTC8163; NZ_LR134439.1; G4T10, NZ_CP011860.1; SE38; NZ_CP011861.1; ZJ, NZ_CP041995.1). The samples were analyzed after a *de novo* assembly with the SKESA (version 2.3.0) algorithm (Souvorov et al., 2018).

Screening for the presence of antimicrobial resistance genes and point mutations causing antimicrobial resistance was performed using Resfinder V4.1 (Zankari et al., 2012), PointFinder (Zankari et al., 2017), and ResFinderFG V1.0 (Sommer et al., 2009; Pehrsson et al., 2016).

## Results and discussion

### Microbiological results and clinical course

*E. rhusiopathiae* was cultured from the wound biopsy. The microbial species was identified using both VITEK^®^ 2 GP ID card with 98% likelihood and MALDI ToF/Vitek MS (99.9%). Susceptibility testing using MIC test strips revealed the minimum inhibitory concentrations (MIC) listed in **Table 1**. For rarely isolated species such as *E. rhusiopathiae*, no specific breakpoints currently exist according to the EUCAST (European Committee on Antimicrobial Susceptibility Testing) guidelines, therefore the interpretation of MIC values was according to the non-species-specific EUCAST PK-PD breakpoints. Following the EUCAST guidance document for the use of PK-PD breakpoints, the results of susceptibility testing cannot be reported in a categorical terms, but only in the form of a guidance for treatment. Accordingly, antimicrobials listed as S “may be used for treatment”, and substances listes as R “should not be used for therapy”. Therefore, due to the non-species-specific EUCAST PK-PD breakpoints, the use of benzylpenicillin, the antibiotic of choice, aminopenicillins cephalosoprines, carbapenems, and fluoroquinolones can be encouraged. In contrast, the U.S. Clinical Laboratory Standards Institute (CLSI) defines *E. rhusiopathiae* specific breakpoints in its document M45 “Methods for Antimicrobial Dilution and Disk Susceptibility Testing of Infrequently Isolated or Fastidious Bacteria”, according to which benzylpenicillin, ampicillin, ceftriaxone, meropenem, imipenem, ciprofloxacin, and levofloxacin have been tested susceptible (**Table 1**). Other potential therapy options due to the CLSI breakpoints were macrolides (such as erthromycin) and clindamycin. However, no EUCAST PK-PD breakpoints are defined for these antimicrobials. The (intrinsic) resistance to vancomycin and aminoglycosides described in the literature was confirmed in the measured MIC values.

**Table 1:** Susceptibility of *E. rhusiopathiae* 319078 to various antimicrobials and assessment according to EUCAST PK-PD breakpoints.

Antimicrobial substance	MIC [mg/L]	Susceptibility EUCAST PK-PD	Susceptibility CLSI
benzylpenicillin	0.032	S	S
ampicillin	0.064	S	S
cefuroxime	0.032	S	–
ceftriaxone	0.032	S	S
ceftazidime	0.032	S	–
meropenem	<0.016	S	S
imipenem	0.008	S	S
ciprofloxacin	0.032	S	S
levofloxacin	0.032	S	S
moxifloxacin	0.032	S	–
gentamicin	128.00	R	R
vancomycin	32.00	IE (R)	R
erthromycin	0.25	IE	S
clindamycin	0.125	IE	S

Postoperative therapy was initial administration of cefazolin 2 g intravenously, followed by ampicillin/sulbactam (Unacid) orally (375 mg q6h) for 5 days (**Table 2**). The follow-up treatment was according to the standard of the “University Clinic for Plastic and Hand Surgery”. The intraoperative wound flap was removed on the first postoperative day. By the fifth postoperative day, there was complete regression of redness, including lymphangitis and swelling. The skin in the wound area appeared non-irritant with contiguous wound edges and splint immobilization of the thumb was terminated. The suture material was removed on the 14^th^ postoperative day.

**Table 2:** Disease progression timeline.

Day	Symptoms, signs, medical findings and procedures
1	presentation with swelling, redness, and pain on the right thumb immediate surgical exploration and tissue biopsy perioperative prophylaxis with cefazolin 2 g i.v.
2	removal of the intraoperative wound flap prescription of ampicillin/sulbactam (Unacid) orally (375 mg q6h) for 5 days
4	cultural detection of *E. rhusiopathiae*
5	availability of the results of the susceptibility testing see **Table 1**
6	complete regression of redness, lymphangitis, and swelling termination of splint immobilization of the thumb ending antibiotic therapy
15	removal of suture material

The origin of the pathogen could not be clarified with complete certainty. The patient denied any direct contact to pigs, including handling of raw pork. She suspected that the source of infection could be a pond in which pigs usually bathe. The pond had no connection to other waters. It was located near several farms and functioned as a watering and bathing place for a number of animals. Fish that could potentially be another source of *E. rhusiopathiae* were not observed in the water body and it is also very unlikely that this water body was a suitable habitat for fish. Her dog bathed in the pond and she petted and dried him afterwards. We attempted to culture *E. rhusiopathiae* from pond water unsuccessfully.

### Genome analysis

SKESA analysis of the Illumina sequences resulted in 50 continuous long reads (CLR) with an average (total) length of 34,401 base pairs. We additionally sequenced long-reads on a MinION (Oxford Nanopore technologies ltd., Oxford, United Kingdom), and performed hybrid assembly with unicycler (v0.4.8, https://github.com/rrwick/Unicycler). This resulted in a single polished contig of 1,780,614 base pairs. The G+C content of the contig was 36.5% and the top species match identity by Ridom Seqsphere+ was *E. rhusiopathiae* by 99%. No extrachromosomal elements were detected.

Application of the NCBI-annotation pipeline resulted in 1,714 genes. Of this total number of genes, 1,621 are protein-coding genes, 13 are pseudo-genes, and 80 are RNA-coding genes. Of the 80 RNA-coding genes, 55 encode tRNAs, 4 encode ncRNAs, 7 encode 5S rRNAs, 7 encode 16S rRNAs, and 7 encode 23S rRNAs. The genome harboured neither prophages nor transposable elements.

**Figure 2:**
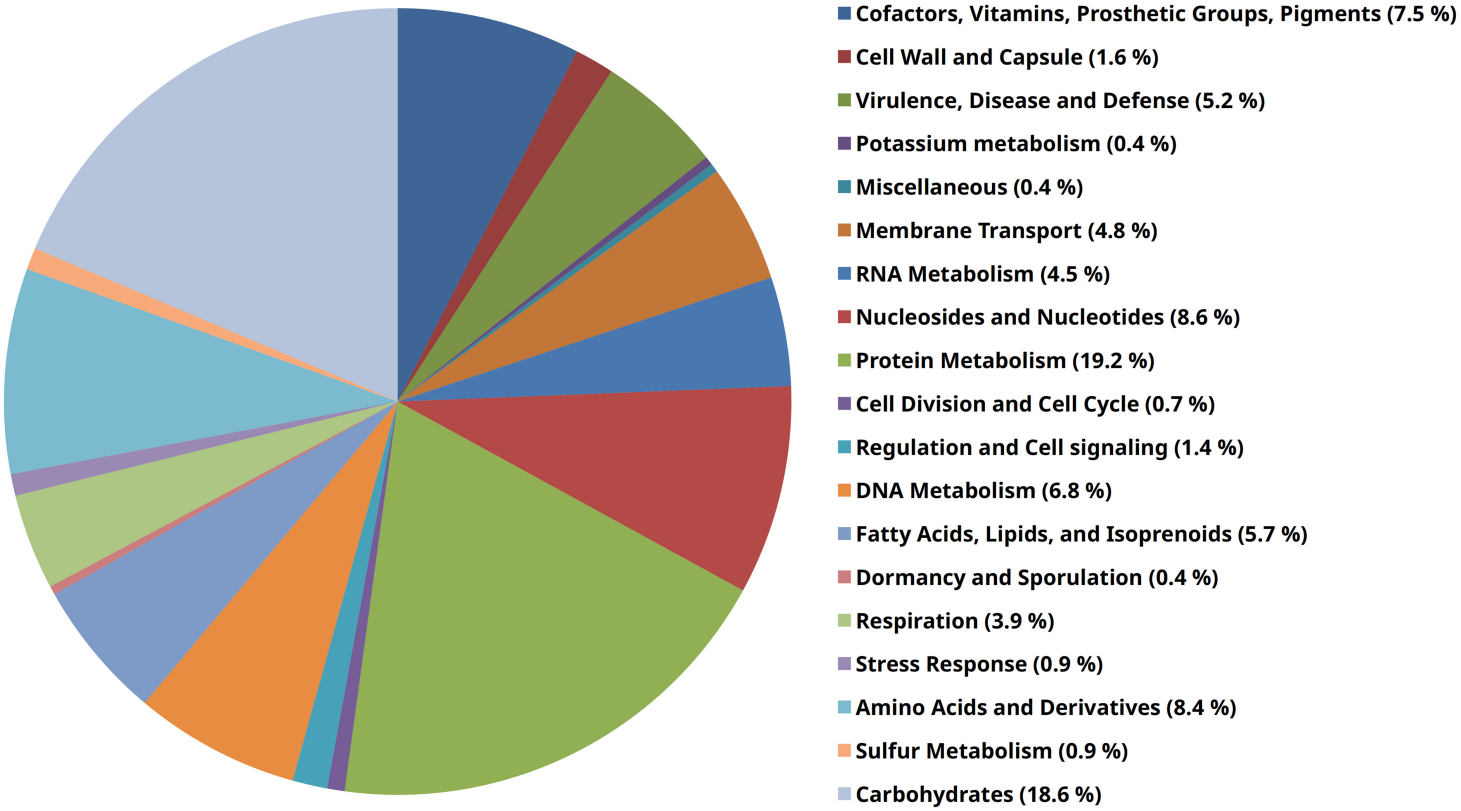
Pie chart of RAST subsystems that were identifiable in the genome of *E. rhusiopathiae* 319078. The 19 most abundant subsystems at the **“**category**”** level identified by RAST are represented by a specific color in the legend on the right side of the figure.

Additional application of the RAST-annotation pipeline yielded 1152 predicted coding sequences and 508 predicted hypothetical proteins. Based on sequence identity, motif analysis, and structural homology to proteins of known function (mostly from other microbial species), a functional prediction of protein function is made as part of the annotation process, which usually requires further experimental verification. The functional categorization of the predicted coding sequences is based on a pure *in silico* analysis that would have to be confirmed *in vitro* or *in vivo* to be considered certain. The RAST subsystem coverage was 25% (413 of 1660 genes). “Protein metabolism” (107 of 558 terms, 19.2%), “carbohydrate metabolism” (104 terms, 18.6%), “nuceloside/nucleotide synthesis” (48 terms, 8.6%), “amino acid metabolism” (47 terms, 8.4%), and “Cofactors, Vitamins, Prosthetic Groups, Pigments” (42 terms, 7.5%) form the largest functional categories in terms of number (**Figure 2**). In order to classify this subsystem category distribution, the distribution of our human isolate was compared to a bovine, a dolphin and a porcine isolate (**Table 3**). However, it must be taken into account that the porcine isolate was also present as a closed genome (1 contig), the dolphin isolate was present as an incomplete genome consisting of 109 contigs, and the bovine isolate genome consisted of 240 contigs from a bovine ruminal metagenome project. Comparison showed that the subsystem category distributions were nearly identical in the human, the porcine, and the dolphin isolate. In the dolphin isolate, one category stood out in comparison to the human and the porcine isolate: “phages, prophages, transposable elements, plasmids”. The genome of the dolphin isolate contained a temperate phage, as indicated by the corresponding genes for a phage terminase, a phage portal protein, a phage-associated type III restriction enzyme, and various bacteriophage hypothetical proteins, among others. The incomplete *E. rhusiopathiae* genome of bovine origin diverged most significantly from the genomes of the other three isolates in subsystem category distribution (**Table 3**). However, these discrepancies were most likely due to the fact that the contigs were derived from a metagenome analysis.

**Table 3:** Comparison of functional subsystem category distribution of *E. rhusiopathiae* isolates of different origins.

Subsystem Category	319078	RUG14096	19DISL	NCTC8163
	human	cattle	dolphin	swine
subsystem coverage	25%	26%	23%	25%
cofactors, vitamins, prosthetic groups, pigments	7.5%	8.2%	7.7%	7.6%
cell wall and capsule	1.6%	7.3%	1.6%	1.6%
virulence, disease and defense	5.2%	4.3%	5.2%	5.2%
potassium metabolism	0.4%	0.4%	0.4%	0.4%
miscellaneous	0.4%	0.5%	0.4%	0.4%
membrane transport	4.8%	2.5%	4.8%	4.9%
RNA metabolism	4.5%	4.5%	4.5%	4.5%
nucleosides and nucleotides	8.6%	6.0%	8.6%	8.7%
protein metabolism	19.2%	8.9%	18.9%	19.3%
cell division and cell cycle	0.7%	0.4%	0.7%	0.7%
regulation and cell signaling	1.4%	0.9%	1.4%	1.4%
DNA metabolism	6.8%	5.0%	6.4%	6.5%
fatty acids, lipids, and isoprenoids	5.7%	2.6%	5.7%	5.8%
dormancy and sporulation	0.4%	0.1%	0.4%	0.4%
respiration	3.9%	3.0%	3.9%	4.0%
stress response	0.9%	1.5%	0.9%	0.9%
amino acids and derivatives	8.4%	19.2%	8.4%	8.5%
sulfur metabolism	0.9%	0.4%	0.9%	0.9%
carbohydrates	18.6%	22.4%	18.9%	18.4%
secondary metabolism	0.0%	0.5%	0.0%	0.0%
nitrogen metabolism	0.0%	1.3%	0.0%	0.0%
phages, prophages, transposable elements, plasmids	0.0%	0.0%	0.4%	0.0%

### Antimicrobial resistance genes

Analysis of the genome using Resfinder V4.1, PointFinder, and ResFinderFG V1.0 (Selected %ID threshold 50% & Selected minimum length 40% for both tools) did not reveal any acquired antimicrobial resistance genes. Genes encoding gyrase (*gyrA*/*B*) and topoisomerase IV (*parC*/*parE*) were identified as potential determinants of quinolone resistance, but since no quinolone resistance was detected phenotypically, it was assumed that these genes were present in the wild-type form and did not contain point mutations that cause quinolone resistance. Furthermore, the genome of *E. rhusiopathiae* 319078 contained three genes encoding for proteins with homology to a MATE (Multidrug And Toxic Compound Extrusion) family MDR efflux pump (**Table 4**). This family of multidrug efflux transporter pumps was associated with fluoroquinolone resistance in *Bacteroides thetaiotaomicron* (Miyamae et al., 2001) as well as with fluoroquinolone, ethidium, and aminoglycoside resistance in *Vibrio parahaemolyticus* (Morita et al., 1998). However, the presence of these genes encoding proteins with a predicted function of MATE family efflux transporters did not confer phenotypic quinolone resistance but could be a factor accounting for the phenotypically observed aminoglycoside resistance in *E. rhusiopathiae* 319078.

**Table 4:** Antimicrobial resistance genes.

Locus tag	Gene	Predicted function
NBX27_04295	*vex3*	ABC transporter membrane-spanning permease
NBX27_04300	*vex2*	ABC transporter, ATP-binding protein
NBX27_05510		MATE family efflux transporter
NBX27_06310		MATE family efflux transporter
NBX27_06335		MATE family efflux transporter
NBX27_00250		beta-lactamase class C-like and penicillin binding proteins (PBPs) superfamily
NBX27_04735		MBL fold metallo-hydrolase, beta-lactamase domain protein
NBX27_07960		MBL fold metallo-hydrolase, Zn-dependent hydrolase (beta-lactamase superfamily)

**Figure 3:**
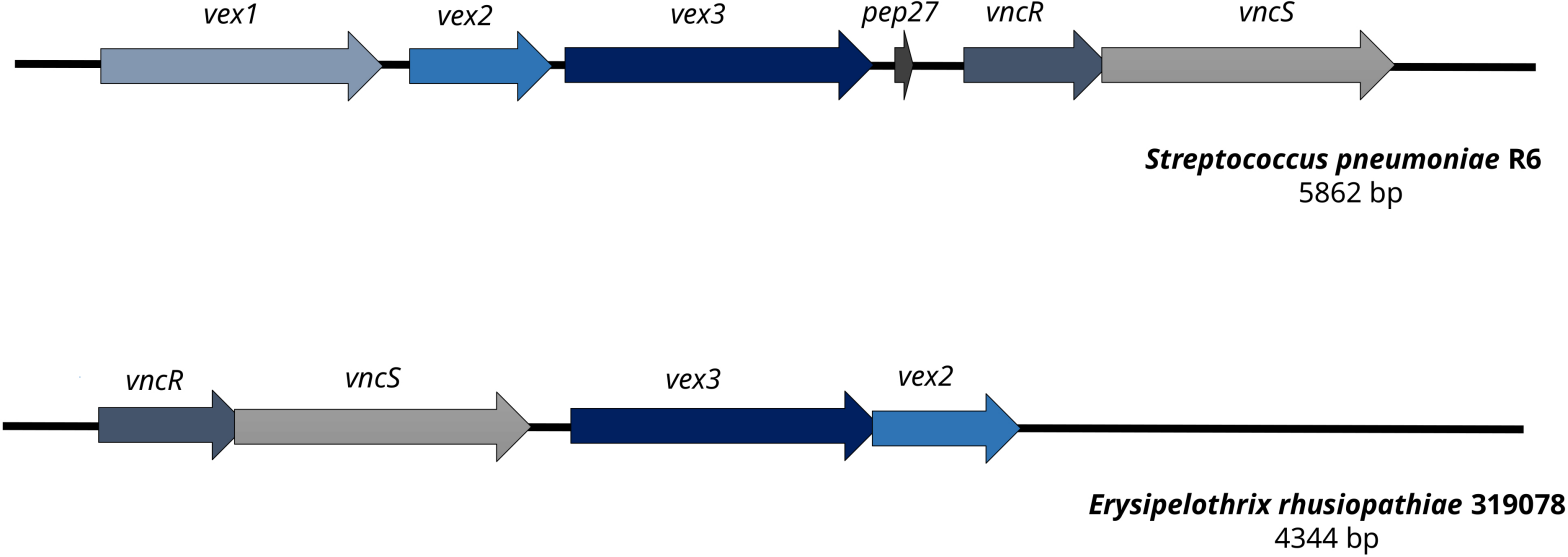
Comparison of the vancomycin tolerance locus of *Streptococcus pneumoniae* R6 and *Erysipelothrix rhusiopathiae* 319078. In contrast to the vancomycin tolerance locus of *S. pneumoniae* R6, *E. rhusiopathiae* 319078 lacks the *pep27* and *vex1* genes, yet the microbial isolate has a vancomycin MIC of 32.0 mg/L.

A gene cluster homologous to the *“Streptococcus pneumoniae* vancomycin tolerance locus” was identified as a presumptive factor for intrinsic vancomycin resistance/tolerance in *E. rhusiopathiae*. The “*Streptococcus pneumoniae* vancomycin tolerance locus” (*vex123*-*pep*_27_-*vncRS* locus) consists of an ABC transporter formed by the gene products of *vex1*, *vex2*, and *vex3*, the two-component response regulator VncR and it’s associated sensor histidine kinase VncS as well as Pep27, a secreted peptide sensed by VncR/S (Novak et al., 1999; Mitchell and Tuomanen, 2002). In contrast, the homologous gene cluster of *E. rhusiopathiae* 319078 lacked the homologue of the secreted peptide gene *pep27* and the homologous gene to the Vex1 subunit of the ABC transporter, a transmenbrane protein (**Figure 3** and **Tables 4**, **5**). While it has been shown that knock-out of *pep27* has no effect on vancomycin-induced autolysis of *S. pneumoniae* (Robertson et al., 2002; Haas et al., 2004), the role of Vex1 has not yet been investigated in detail, neither in *S. pneumoniae* nor in *E. rhusiopathiae*. Vex1 and Vex3 are proposed to form a transmembrane protein channel while Vex2 is an ATP-binding cassette protein. It may be that in *E. rhusiopathiae* the Vex3 homolgue alone would able to shape a sufficient transmembrane channel. Since vancomycin resistance of *E. rhusiopathiae* is considered a characteristic resistance of this microbial species, further investigation of this four-gene gene cluster provides an interesting starting point for future experiments on vancomycin resistance. On the other hand, this gene cluster may also be completely non-functional, mainly due to the absence of a gene with predicted function of the Vex1 protein. The *vex23*-*vncRS* locus of *E. rhusiopathiae* is highly conserved in the available genome sequences. Among the 10 *E. rhusiopathiae* genomes deposited at NCBI, *vex2* has 99.84% to 100% sequence identity at a 100% coverage, *vex3* has 99.64% to 100% sequence identity at a 100% coverage, *vncS* has 99.64% to 99.86% sequence identity at a 100% coverage, and *vncR* (*luxR*) has 99.55% to 100% sequence identity at a 99% to 100% coverage.

**Table 5:** Enzymes involved in peptidoglycan biosynthesis.

Locus tag	Gene	Predicted function
NBX27_08175	*murA*	UDP-N-acetylglucosamine-1-carboxyvinyltransferase
NBX27_02770	*murB*	UDP-N-acetylmuramate dehydrogenase
NBX27_02935	*murC*	UDP-N-acetylmuramate–L-alanine ligase or UDP-N-acetylmuramate–L-serine ligase?
NBX27_02805	*murD*	UDP-N-acetylmuramoyl-L-alanine-D-glutamate ligase
NBX27_04865	*murE*	UDP-N-acetylmuramoylalanyl-D-glutamate–L-alanine ligase
NBX27_03210	*murF*	UDP-N-acetylmuramoyl-tripeptide–D-alanyl-D-alanine ligase
NBX27_02800	*mraY*	Phospho-N-acetylmuramoyl-pentapeptide-transferase
NBX27_04480	*murG*	Undecaprenyldiphospho-muramoylpentapeptide beta-*N*-acetylglucosaminyltransferase
NBX27_05455	*murI*	glutamate racemase
NBX27_05555	*murJ/mviN*	murein biosynthesis integral membrane protein MurJ
NBX27_00365	*alr*	alanine racemase
NBX27_00660	*glmM*	phosphoglucosamine mutase
NBX27_02455	*glmS*	glutamine–fructose-6-phosphate transaminase
NBX27_02525	*mltG*	endolytic transglycosylase MltG
NBX27_03160	*uppS*	polyprenyl diphosphate synthase
NBX27_08340	*glmU*	bifunctional UDP-*N*-acetylglucosamine diphosphorylase/glucosamine-1-phosphate N-acetyltransferase GlmU
NBX27_02795	*pbp*	penicillin-binding protein

In addition, one gene encoding a protein with the predicted function of a class C beta-lactamase and two genes each encoding a protein with the predicted function of a metallo-beta-lactamase were also present in the genome of *E. rhusiopathiae* 319078 (**Table 4**). However, phenotypically, all penicillins, aminopenicillins, cephalosporins, and carbapenems were tested susceptible.

### Peptidoglycan biosynthesis

*E. rhusiopathiae* possesses a complete set of genes encoding enzymes with a predicted function for peptidoglycan biosynthesis (**Table 5**). These genes are not organized in a cluster but are scattered throughout the whole genome. In previous writings, intrinsic vancomycin resistance of this microbial species was thought to be due to the termination of the peptide stem of the peptidoglycan with D-alanine-D-lactate. This would be similar to what was found in vancomycin-resistant enterococci of the *vanA*/*vanB* phenotype (Nelson, 1999). However, according to our annotation, this does not seem to be the case, since the predicted function of the *murF* gene (NBX27_03210) was a UDP-N-acetylmuramoyl-tripeptide–D-alanyl-D-alanine ligase, and thus the pentapeptide stem ending would be D-Ala-D-Ala. Nevertheless, *E. rhusiopathiae* has some peculiarities concerning the cell wall peptidoglycan structure. There was a discrepancy in the predicted function of the protein encoded by *murC* between the NCBI and RAST annotation piplines. While NCBI predicted here the function of a UDP-N-acetylmuramate–L-alanine ligase the predicted function according to RAST was UDP-N-acetylmuramate-L-serine ligase. Thus, instead of an L-Ala, there could be an L-Ser at position 1 of the pentapeptide as in *Butyribacterium rettgeri* (Vollmer et al., 2008). A second peculiarity exists at position 3 of the pentapeptide. Instead of an L-Lys, as found in most Gram-positive bacteria, *E. rhusiopathiae* has a D-Ala at this position (Vollmer et al., 2008), which was also in agreement with the predicted function of the *murE* gene product as a UDP-N-acetylmuramoylalanyl-D-glutamate-L-alanine ligase. The pentapeptide stem of *E. rhusiopathiae* therefore should have an amino acid sequence of D-Ala-D-Glu-D-Ala-D-Ala-D-Ala or possibly L-Ser-D-Glu-D-Ala-D-Ala-D-Ala. To what extent this specific structure of the pentapeptide is related to the intrinsic vancomycin resistance of the bacterium requires further investigation.

### Virulence factors

The virulence factors of *E. rhusiopathiae* were systematically characterized as part of the first complete genome announcement by Ogawa and colleagues (Ogawa et al., 2011). After more than a decade of improvements in the annotation pipelines and since our isolate was of human origin we decided to reassess the virulence factors in the genome of our isolate.

#### Two-component signal transduction systems

Bacteria regulate the expression of a variety of genes, including those encoding virulence-associated factors, with two-component signal transduction systems integrating external signals. Ogawa and coworkers were able to identify a total of 15 genes that presumptively encode response regulators, and for 14 of them they were able to identify the corresponding presumptive sensor histidine kinase upstream or downstream. But unfortunately, they could assign a predicted function only for 4 two-component signal transduction systems (Ogawa et al., 2011). With the help of the annotation pipelines we employed (NCBI & RAST), we were able to assign a predicted function to all 14 two-component signal transduction systems (**Table 6**). As shown previously by Ogawa and coworkers, the two-component system NBX27_00670/NBX27_00665 exhibited homology to CssS & CssR and therefore its presumptive function could be the control of cellular responses to protein secretion stress (Hyyryläinen et al., 2001; Ogawa et al., 2011). Similarly, the two-component system, for which a function in the regulation of the phosphate regulon responsible for uptake of inorganic phosphate was predicted, had also been localized in the genome of *E. rhusiopathiae* 319078 (NBX27_04520/NBX27_04525) (Ogawa et al., 2011; Santos-Beneit, 2015). Due to their sequence identity, we could assign two further two-component systems (NBX27_01470/NBX27_01465 & NBX27_06020/NBX27_06025) to the LytTR family, which are potentially involved in regulating the expression of many virulence factors, e.g. extracellular polysaccharides, toxins and bacteriocins (Nikolskaya and Galperin, 2002; Ogawa et al., 2011). Three of the two-component systems (NBX27_01190/NBX27_01185, NBX27_07145/NBX27_07140, NBX27_07455/NBX27_07460) had response regulators with homologues to the LuxR family and could presumptively be involved in the regulation of quorum-sensing factors. The ComD/ComE system was shown to function as a negative transcriptional regulator of the capsular polysaccharide (*cps*) locus of *S. pneumoniae (* Zheng et al., 2017 *)*. A pair of homologous genes (NBX27_03955/NBX27_03960) was detected in *E. rhusiopathiae* 319078. The two-component signal transduction system NBX27_04290/NBX27_04285, which was homologous to *vncS*/*vncR* of *S. pneumoniae*, has already been discussed in the subsection “Antimicrobial resistance genes”, as it may presumptively play a role in tolerance to vancomycin *via* regulation of *vex2* & *vex3* expression. Furthermore, we could localize two-component signal transduction systems in the genome of *E. rhusiopathiae* 319078 for which a function in the regulation of thioredoxin reductase expression, adaptation to osmolality, invasin expression, as well as magnesium and cobalt transport was predictable (**Table 5**). In the vicinity of the response regulator localized at locus tag NBX27_06540, no sensor histidine kinase was found in the genome of *E. rhusiopathiae* 319078, so that this remains an orphan response regulator gene (**Table 6**).

**Table 6:** Two-component signal transduction systems.

Kinase	Response regulator	Predicted function
NBX27_00670	NBX27_00665	CssS, HAMP domain-containing histidine kinase & CssR, response regulator transcription factor (control of cellular responses to protein secretion stress)
NBX27_01190	NBX27_01185	HAMP domain-containing histidine kinase & LuxR family, response regulator transcription factor (quorum-sensing)
NBX27_01280	NBX27_01285	sensor histidine kinase, YesM & response regulator transcription factor, TrxR (thioredoxin reductase)
NBX27_01470	NBX27_01465	GHKL domain-containing protein & LytTR family DNA-binding domain-containing protein (virulence factors, e.g. extracellular polysaccharides, toxins and bacteriocins)
NBX27_01630	NBX27_01625	ATP-binding protein & response regulator transcription factor, OmpR family (adaptation to osmolality in *E. coli*; invasin expression in *Yersinia enterocolitica* (Brzóstkowska et al., 2012))
NBX27_01645	NBX27_01640	HAMP domain-containing histidine kinase & response regulator transcription factor, YrkP (Ogura et al., 2008)
NBX27_03955	NBX27_03960	histidine kinase of the competence regulon ComD & response regulator of the competence regulon ComE (capsular polysaccharide, CPS)
NBX27_04290	NBX27_04285	VncS, HAMP domain-containing sensor histidine kinase & VncR-homologue, two-component transcriptional response regulator (vancomycin tolerance)
NBX27_04520	NBX27_04525	two-component system sensor histidine kinase & phosphate regulon transcriptional regulatory protein PhoB (SphR, Pi uptake)
NBX27_04825	NBX27_04830	osmosensitive K^+^ channel histidine kinase KdpD & two-component transcriptional response regulator, OmpR family (adaptation to osmolality)
NBX27_05995	NBX27_06000	ABC transporter-like sensor linked histidine kinase & ABC transporter-like sensor linked response regulator (magnesium and cobalt transport)
NBX27_06020	NBX27_06025	GHKL domain-containing protein & LytTR family DNA-binding domain-containing protein (virulence factors, e.g. extracellular polysaccharides, toxins and bacteriocins)
–	NBX27_06540	orphan response regulator
NBX27_07145	NBX27_07140	sensor histidine kinase & two-component transcriptional response regulator, LuxR family (quorum-sensing)
NBX27_07455	NBX27_07460	ABC transporter-coupled two-component system, signal transduction histidine kinase & ABC transporter-coupled two-component system, LuxR family response regulator (quorum-sensing)

#### Capsular polysaccharide synthesis

Another important virulence-associated factor is the ability of a bacterium to form a capsule, or capsular polysaccharide synthesis. A seven-gene capsular polysaccharide synthesis locus was identified in the genome of *E. rhusiopathiae* Fujisawa (Ogawa et al., 2011), which was also found in *E. rhusiopathiae* 319078 at 100% coverage and 99.24% sequence identity.

#### Surface-associated proteins

In the genome of *E. rhusiopathiae* Fujisawa, a total of 21 proteins was detected containing an LPTXTG-motif. Based on this motif, these proteins are predicted to be covalently linked to peptidoglycan chains by a specific sortase, and based on this pepdidoglycan linkage, it is assumed that these were surface-associated proteins (Ogawa et al., 2011). Both the sortase (NBX27_00075) and its potential 21 substrates were localized by us in the genome of *E. rhusiopathiae* 319078. Updates in the annotation are provided in **Table 7**. Of particular note is the surface protective antigen adhesin SpaA (NBX27_00545), which also functions as antigen in many subunit vaccines (Opriessnig et al., 2020). SpaA itself belongs to three surface proteins that bind to choline residues of teichoic acid and by this become membrane anchored (Ogawa et al., 2011; Borrathybay et al., 2015). It plays a significant role in virulence, adhesion to host cells, and serum resistance of *E. rhusiopathiae* (Borrathybay et al., 2015). In addition, two homologues to the *Streptococcus pyogenes* shaft pilin SpaA (Ramirez et al., 2020) were found in the genome (NBX27_00445 & NBX27_07175), which should not be confused with the surface protective antigen adhesin SpaA of *E. rhusiopathiae* and which possess an LPXTG motif and are therefore predicted to be membrane-anchored *via* the sortase already described. Hyaluronate lyases are considered to be a significant virulence factor, especially with regard to spreading in relatively hyaluronic acid-rich tissues such as the skin. Three coding sequences (CDSs) encoding proteins for which a hyaluronate lyase activity was predicted (NBX27_00835, NBX27_03750, & NBX27_06110) were found in the genome of *E. rhusiopathiae* 319078, representing potentially important factors in the pathogenesis of erysipeloid. Major virulence factors with complex action are bacterial neuraminidases (Soong et al., 2006). Both the *E. rhusiopathiae* Fujisawa (Ogawa et al., 2011) and *E. rhusiopathiae* 319078 genomes have two CDSs encoding for proteins for which a neuraminidase function was predicted. One carries the LPXTG motif and therefore should potentially be cell surface associated (NBX27_01575), the second apparently could act potentially as an extracellular enzyme (NBX27_03725).

**Table 7:** Bacterial surface proteins.

Locus tag	Gene	Predicted function
NBX27_00075		(sortase A, LPXTG specific)
NBX27_00445		shaft pilin (SpaA) isopeptide-forming pilin-related protein
NBX27_00545	*spaA*	surface protective antigen adhesin SpaA (choline-binding protein)
NBX27_00835	*hylA*	hyaluronate lyase precursor, polysaccharide lyase, family 8
NBX27_00890		LPXTG cell wall anchor domain-containing protein, peptidase M14
NBX27_01135		InlB B-repeat-containing protein
NBX27_01240		family 16 glycosylhydrolase, sialidase
NBX27_01430		DUF4573 domain-containing protein, cell-envelope associated proteinase, subtilase family
NBX27_01495		LPXTG cell wall anchor domain-containing protein
NBX27_01575	*nanH.1*	exo-alpha-sialidase (neuraminidase)
NBX27_02145	*cbpA*	glucosaminidase domain-containing protein, Choline binding protein A
NBX27_02955		discoidin domain-containing protein
NBX27_03280		Cna B-type domain-containing protein
NBX27_03285		Cna B-type domain-containing protein
NBX27_03565		leucine-rich repeat domain-containing protein, possible surface protein responsible for cell interaction; contains cell adhesion domain and ChW-repeats
NBX27_03750	*hylB*	hyaluronate lyase precursor, polysaccharide lyase, family 8
NBX27_03765	*cbpB*	choline-binding protein
NBX27_03810		C69 family dipeptidase
NBX27_05710	*ushA*	5’-nucleotidase C-terminal domain-containing protein
NBX27_06110	*hylC*	hyaluronate lyase precursor, polysaccharide lyase, family 8
NBX27_06345		cell wall anchor protein
NBX27_07175		(shaft pilin) SpaA isopeptide-forming pilin-related protein
NBX27_07275		putative peptidoglycan bound protein (LPXTG motif) Lmo2179 homolog, peptidase
NBX27_07355		protein phosphatase 1 regulatory subunit 42
NBX27_08485		Cna B-type domain-containing protein, LPXTG-motif cell wall anchor domain

#### Inactivation of reactive oxygen species

Other significant virulence-associated factors are those that enable intracellular survival of the bacterium. Bacteria must protect themselves from reactive oxygen species (ROS) after the formation of the phagolysosome. Analysis of the *E. rhusiopathiae* Fujisawa genome identified 9 genes encoding enzymes with a predicted function indicating that they potentially play a role in the neutralization of ROS: a predicted superoxide dismutase, two predicted thioredoxins, two predicted thioredoxin-disulfide reductases, a predicted thiol peroxidase, a predicted glutaredoxin, and two predicted alkylhydroperoxide reductases (Ogawa et al., 2011). We were able to add three more CDSs to this funcional subgroup (**Table 8**): a predicted third thioredoxin gene (NBX27_00960), a predicted peptide methionine (S)-S-oxide reductase MsrA (NBX27_00585) that presumably reduces ROS-generated methionine sulfoxide in proteins back to methionine (Weissbach et al., 2002), and a predicted peroxide stress protein YaaA-homologue (NBX27_07905). YaaA was shown to reduce hydrogen peroxide induced damage by decreasing the fraction of intracellular unincorporated iron (Liu et al., 2011).

**Table 8:** Antioxidant factors.

Locus tag	Gene	Predicted function
NBX27_00585	*msrA*	peptide-methionine (S)-S-oxide reductase MsrA
NBX27_00895	*tpx*	thiol peroxidase
NBX27_00955	*ahpC*	peroxiredoxin, Bcp-type
NBX27_00960		thioredoxin
NBX27_01880	*nrdH*	glutaredoxin
NBX27_01975	*trxA.1*	thioredoxin
NBX27_05285	*sodA*	superoxide dismutase
NBX27_06495	*trxB.1*	thioredoxin reductase, NAD(P)/FAD-dependent oxidoreductase
NBX27_06660	*ahpD*	carboxymuconolactone decarboxylase family protein
NBX27_07535	*trxA.2*	thioredoxin
NBX27_07755	*trxB.2*	thioredoxin reductase, FAD-dependent oxidoreductase
NBX27_07905	*yaaA*	peroxide stress protein YaaA

#### Phospholipases

Another group of enzymes that play a role in the intracellular life cycle of some bacteria are phospholipases. For example, it has been shown that patatin phospholipases of *Rickettsia typhi* contribute to open the phagosome or phagolysosome membrane and allow the bacterium to escape into the cytoplasm (Rahman et al., 2013; Smith and May, 2013). More recent studies demonstrated that phospholipases aid in the escape from vacuoles and phagosomes for *Listeria monocytogenes*, *Shigella* spp., *Plasmodium berghei*, *Salmonella* spp., and *Legionella pneumophila* (Bianchi and van den Bogaart, 2020; Petrišič et al., 2021; Srivastava and Mishra, 2022). Additionally, it was experimentally proven that *Mycobacterium tuberculosis* recruits the cytoplasmic phospholipase A_2_ to permeabilize the endosomal membrane in infected macrophages and to translocate to the cytosol (Jamwal et al., 2016). On the other it was shown that *E. rhusiopathiae* predominantly replicates in the cytoplasm of macrophages in the spatial vicinity of the entry site (Shimoji et al., 1996; Shimoji, 2000). Therefore, Ogawa and colleagues postulated that phospholipases also play a pivotal role in intracellular translocation of *E. rhusiopathiae* during phagosome opening. However, experimental evidence of this role remains to be provided for this microbial species. Ogawa and coworkers identified a total of 9 CDSs with homology to phospholipases in the genome of *E. rhusiopathiae* Fujisawa genome (Ogawa et al., 2011), which we also found in the genome of *E. rhusiopathiae* 319078 (**Table 9**).

**Table 9:** Phospholipase genes.

Locus tag	Gene	Predicted function
NBX27_00385		patatin-like phospholipase family protein
NBX27_00485		dienelactone hydrolase family protein
NBX27_00825	*pldB*	lysophospholipase, monoglyceride lipase
NBX27_01735	*cls*	cardiolipin synthase
NBX27_01740		patatin family protein
NBX27_01835		dienelactone hydrolase family protein
NBX27_02035		phospholipase D family protein
NBX27_06130		lysophospholipase, monoglyceride lipase alpha/beta hydrolase
NBX27_07160		lysophospholipase

#### Further virulence associated factors

One of the predicted virulence-associated factors additionally detected by RAST subsystem analysis in the genome of *E. rhusiopathiae* 319078 was a homologue to the multiple sugar metabolism regulator, (MsmR, NBX27_01505, **Table 10**). MsmR, an AraC/XylS type transcriptional regulator, is part of the *Streptococcus pyogenes* recombinatorial zone. In *S. pyogenes* this highly recombinatorial zone consists of genes encoding chaperonin, Hsp33; sortase, Spy0135; serum opacity factor, SOF; transcriptional regulator, RofA; negative transcriptional regulator, Nra; fibronectin-binding protein, PrtF; fibronectin-binding protein 2, PrtF2; collagen-binding adhesin, Cpa; multiple sugar metabolism regulator, MsmR; electron transfer flavoprotein 1A, EtfLS; and signal peptidase I, LepL. The gene products include several MSCRAMMs (microbial surface components recognizing adhesive matrix molecules) and play a crucial role in pili-production and mediate adhesion to human cells and tissues (Podbielski et al., 1999; Kreikemeyer et al., 2007). Transcriptome analysis in *S. pyogenes* serotype M49 showed that the MsmR regulon contains 24 genes under positive MsmR control and 36 genes repressed by MsmR (Nakata et al., 2005). It was demonstrated by electrophoretic mobility shift assay (EMSA) that MsmR binds directly to the promoter regions of the genes encoding fibronectin-binding protein 2 (*prtF2*), negative regulator of group A Streptococci (*nra*), collagen-binding protein (*cpa*), NAD-glycohydrolase (*nga*), and streptolysin O (*slo*) (Nakata et al., 2005). In Gram-positive bacteria such as group A streptococci, a cytolysin-mediated translocation (CMT) system replaces the “type III secretion machinery” commonly found in Gram-negative bacteria. Transcription of this CMT system, which plays an important role in host cell interaction, is regulated in particular by MsmR (Madden et al., 2001). However, only a MsmR homologue of this gene cluster is present in the genome of *E. rhusiopathiae* 319078. Whether a similar role in host cell adherence, internalization, and cytotoxicity exists in *E. rhusiopathiae* analogous to the role in *S. pyogenes* remains to be confirmed experimentally. The predicted MsmR was detectable in all of the 10 *E. rhusiopathiae* genomes deposited at NCBI with 99.84-99.92% sequence identity at a 100% coverage. Therefore, the MsmR homologue appears to be ubiquitous in *E. rhusiopathiae*. In addition, genes for a predicted type III hemolysin (NBX27_03180) and a predicted hemolysin-related protein (NBX27_02485) with a cystathionine-beta-synthase (CBS) domain exist in the *E. rhusiopathiae* 319078 genome that could be part of a potential CMT system. Another predicted hemolysin not previously described for *E. rhusiopathiae* is a homologue of the thermostable hemolysin delta-VPH (NBX27_08265), which was first described in *Vibrio parahaemolyticus* (Taniguchi et al., 1990).

**Table 10:** Virulence assocated factors (incl. hemolysins, adhesins, etc.).

Locus tag	Gene	Predicted function
NBX27_03725	*nanH.2*	glycoside hydrolase (neuraminidase)
NBX27_02485		hemolysin family protein (containing CBS domains)
NBX27_03180		hemolysin III family protein
NBX27_08265		thermostable hemolysin delta-VPH
NBX27_01505		multiple sugar metabolism regulator (MsmR), AraC family transcriptional regulator
NBX27_05085		fibronectin/fibrinogen-binding (NFACT family) protein
NBX27_06720	*znuA*	zinc ABC transporter substrate-binding protein
NBX27_07335		Ig-like domain-containing protein

## Conclusions

In summary, we presented a patient with erysipeloid and lymphangitis. The site of entry was probably a minor trauma of the thumb. The source of infection could not be identified with certainty. A body of water in which pigs and the patient’s dog bathed consecutively was considered as potential reservoir. The genome of the *E. rhusiopathiae* isolate causative for the infection was whole-genome sequenced. A predicted *vex23-vncRS* locus homologous to the “*S. pneumoniae* vancomycin tolerance locus” and three predicted MATE family efflux transporters were identified as potential antimicrobial resistance determinants. Several genes encoding proteins with predicted functions that qualify them as potential virulence-associated factors have been identified, including a predicted homologue to the multiple sugar metabolism regulator, MsmR, which cloud play a role in host cell interaction, three predicted hemolysins, two predicted neuraminidases, three predicted hyaluronate lyases, the surface protective antigen adhesin SpaA, a subset of predicted enzymes that potentially play a role in intracellular survival, several predicted two-component signal transduction systems, two dozen predicted surface-associated proteins and a homologue to the capsular polysaccharide synthesis locus.

## Data availability statement

The datasets presented in this study can be found in online repositories. The names of the repository/repositories and accession number(s) can be found below: https://www.ncbi.nlm.nih.gov/genbank/, CP098031.

## Ethics statement

Ethical review and approval was not required for the study on human participants in accordance with the local legislation and institutional requirements. Written informed consent for participation was not required for this study in accordance with the national legislation and the institutional requirements.

## Author contributions

Conceptualization, AK, AZ; methodology, AK, AZ; writing-original draft preparation, AZ, C-JS; writing-review and editing, AT, C-JS, PM, MR, MÐ, AD, DM, AK, AZ; visualization, AZ, MÐ; investigation, MÐ, DM; funding acquisition AK, whole genome sequencing and genome assembly, AT, PM, MR; data curation, annotation, patient treatment, AD; All authors contributed to the article and approved the submitted version.

## Funding

The research of the authors was funded by the Deutsche Forschungsgemeinschaft (grant number ZA 697/6-1).

## Acknowledgments

We are grateful to Nadja Schlüter, Francis Meier and Sabine Jürgenfeld for excellent technical assistance.

## Conflict of interest

The authors declare that the research was conducted in the absence of any commercial or financial relationships that could be construed as a potential conflict of interest.

## Publisher’s note

All claims expressed in this article are solely those of the authors and do not necessarily represent those of their affiliated organizations, or those of the publisher, the editors and the reviewers. Any product that may be evaluated in this article, or claim that may be made by its manufacturer, is not guaranteed or endorsed by the publisher.
